# 753 Aquatic Therapy Improves Functional Outcomes in a Pediatric Burn Patient

**DOI:** 10.1093/jbcr/irad045.228

**Published:** 2023-05-15

**Authors:** Maria Arango, Carl Schulman

**Affiliations:** University of Miami-Jackson Memorial Hospital, Miami, Florida; University of Miami-Jackson Memorial Hospital, Miami, Florida; University of Miami-Jackson Memorial Hospital, Miami, Florida; University of Miami-Jackson Memorial Hospital, Miami, Florida

## Abstract

**Introduction:**

Burn injuries in the pediatric population require extended recovery time due to changes in their respiratory function and musculoskeletal system (prolonged fascia damages and muscular weakness) leading to low physical and functional capacity. Other external factors contributing to poor outcomes are pain, psychosocial changes such as depression, self-esteem and body image. In the rehabilitation process there are different modalities and techniques for that can be used to enhance recovery. Aquatic therapy is a modality that facilitates movement. The buoyancy of the water supports body weight and reduces the load on painful joints. The density of the water forces the body upwards, the relatively high water temperature promotes muscle relaxation, and the hydrostatic pressure reduces the development of edema. We hypothesized that aquatic therapy would improve motor skill, respiratory function, and musculoskeletal functional capacity in pediatric burn patients.

**Methods:**

This is a case comparison study comparing the functional outcome on a 6 y/o burned patient utilizing aquatic therapy compared with the same age patient that had traditional therapy in a regular play gym. Both patients had second degree burns TBSA 20 to 30% in the arms and face area, requiring autografts from chest/back and legs. The study treatment consisted of intensive interdisciplinary therapy in the pool for 90 minutes, one time per week for 8 months. The therapy involved games with physical demands to improve cardiorespiratory endurance, balance, postural control, body alignment, fine and gross motor skills, and the functional use of full range of motion, strength and dexterity. At completion of the aquatic program, Active Range of Motion measurements of the joints was performed using a goniometer and the Bruininks-Oseretsky Test of Motor Proficiency in order to evaluate functional improvements.

**Results:**

Our results showed that the aquatic program increased motivation, respiratory capacity, achieved full AROM in all joints involved. The score in the Bruininks-Oseretsky Test of Motor Proficiency for each patient is shown in Table 1. It was demonstrated that the patient that did not use aquatic therapy as a modality did not regain full range and function compared to the aquatic therapy patient.

**Conclusions:**

The use of aquatic therapy as a modality in pediatric burn patients can provide direct benefits such as gaining full AROM to participate in fine motor and gross motor functional activities and indirect benefits like motivation due to the engagement to learn to swim, play in the water and community reintegration.

**Applicability of Research to Practice:**

The positive results of aquatics on this pediatric burn patient and the review of other studies support further development of an aquatic program as a positive rehabilitation modality.

